# The Assembling of Poly (3-Octyl-Thiophene) on CVD Grown Single Layer Graphene

**DOI:** 10.1038/srep17720

**Published:** 2015-12-04

**Authors:** Yanqiu Jiang, Ling Yang, Zongxia Guo, Shengbin Lei

**Affiliations:** 1 Key Laboratory of Microsystems and Microstructures Manufacturing, Ministry of Education, Harbin Institute of Technology, Harbin, 150080, People’s Republic of China; 2 School of Polymer Science and Engineering, Qingdao University of Science and Technology, Qingdao, 266042, People’s Republic of China

## Abstract

The interface between organic semiconductor and graphene electrode, especially the structure of the first few molecular layers at the interface, is crucial for the device properties such as the charge transport in organic field effect transistors. In this work, we have used scanning tunneling microscopy to investigate the poly (3-octyl-thiophene) (P3OT)-graphene interface. Our results reveal the dynamic assembling of P3OT on single layer graphene. As on other substrates the epitaxial effect plays a role in determining the orientation of the P3OT assembling, however, the inter-thiophene distance along the backbone is consistent with that optimized in vaccum, no compression was observed. Adsorption of P3OT on ripples is weaker due to local curvature, which has been verified both by scanning tunneling microscopy and density functional theory simulation. Scanning tunneling microscopy also reveals that P3OT tends to form hairpin folds when meets a ripple.

Because of the extremely high electron mobility and optical transparency, graphene has been considered as an excellent candidate for replacing indium tin oxide (ITO) as transparent electrode in devices such as organic field effect transistors (OFETs), organic light-emitting diodes (OLEDs) and flexible displays^1^,^2^. In all these applications understanding the interfacial issues between graphene electrode and organic semiconductors is of great significance for improving the device performance^3^,^4^,^5^. Recently, a series of studies has been carried out focusing on this issue. The Moiré pattern of epitaxial graphene on SiC and metals (Ru, Ir, Rh, Pt) was reported to direct the adsorption and assembly of organic semiconductors, such as phthalocyanine, pentacene and perylene tetracarboxylic diimide (PTCDI)^6^,^7^,^8^,^9^,^10^,^11^,^12^,^13^,^14^,^15^. Graphene grow on SiC or Ru (0001) was also suggested to act as a buffer layer to electronically decouple the adsorbate from the substrate, such as 3, 4, 9, 10-perylene-tetracarboxylic dianhydride (PTCDA), pentacene, and C_60_ molecules, preserving the intrinsic electronic structure of the adsorbed molecules^6^,^7^,^8^. The effect of surface corrugation and defect-like ripple and wrinkle on the assembly of organic semiconductors was also investigated^14^,^15^.

Regio-regular poly(3-alkyl-thiophene)s (P3ATs) are used as active components in plastic electronics, such as polymer solar cells and OFETs^16^,^17^,^18^, because of their high charge carrier mobility, excellent processability and environmental stability. In these devices, the packing structure and side chain arrangement along the conjugated backbone are crucial to their properties, for instance optical properties, conductivity and mobility^18^,^19^. In particular the device properties such as the charge transport in OFETs are governed by the structure of the first few molecular layers at the interface. Thus the assembling behaviour of P3ATs on conductive materials has attracted a great deal of attention^24^. Though the structure and properties of P3AT assemblies have been extensively investigated on HOPG surface^20^,^21^,^22^, graphene is definitely a more attractive substrate considering its potential application as transparent electrode in OLED and polymer solar cells^1^,^2^,^3^,^4^,^5^, and has more significance towards understanding the relation of interfacial structure and device performance^5^,^18^,^19^

In this work we focus on the interfacial structure of poly(3-octylthiophene) (P3OT) on a single layer graphene grown on multi-crystalline copper foil (SLG-copper). The assembling behaviour of P3OT at the solid-liquid interface was monitored *in situ* by scanning tunnelling microscopy. Beside its assembling on flat terraces, we pay special attention to the influence of unique characteristics of chemical vapour deposition (CVD) grown graphene, to name a few, the different facets and step edges of copper substrate, ripples and wrinkles along the boundaries between crystalline and noncrystalline areas or on step edges of crystalline areas^12^,^14^,^15^.

## Results and Discussion

**Figure 1:**
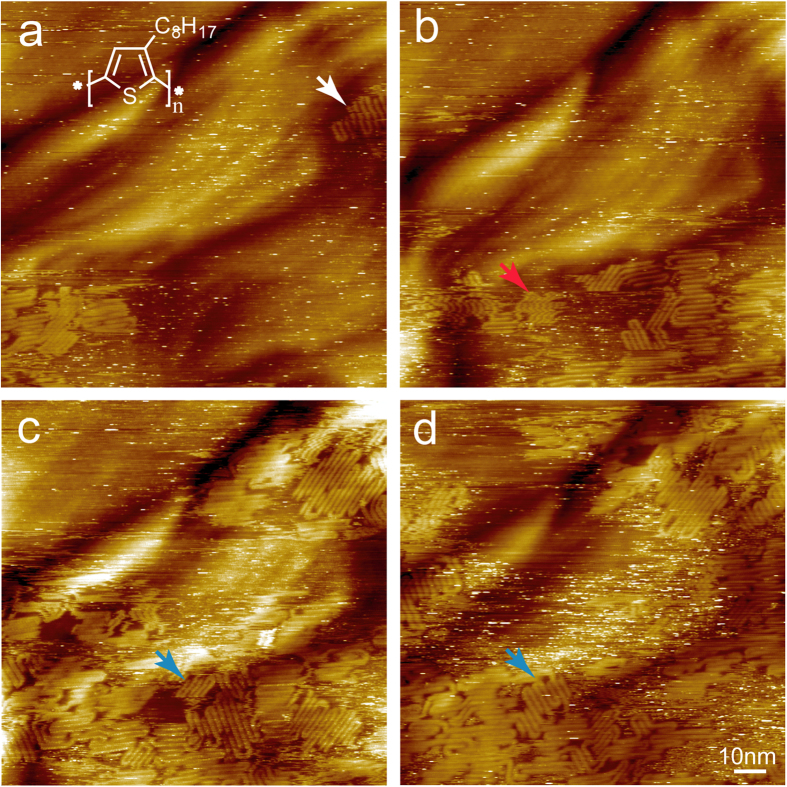
A series of STM images shows the evolution of P3OT adlayer on SLG-copper. A hexagonal superlattice can be seen clearly, which indicates the graphene is grown on a Cu (111) terrace. (**a**) and (**b**) are consecutive images with time interval of 85 sec, and the time interval between (**b**,**c**) and (**c**,**d**) are 11 and 14 min, respectively. The P3OT adlayer appears to be very dynamic, desorption, translation and change of domain orientation are frequently observed as indicated by the white, red and blue arrow heads, respectively. V_bias_ = 1.2 V, I_set_ = 30 pA.

In this work we performed STM characterization at the solid-liquid interface. P3OT was dissolved in phenyloctane with a concentration of 0.1 mg/g and a droplet of this solution was deposited on SLG-copper. As shown in Fig. 1, P3OT starts to adsorb on the surface at areas close to step edges of underneath copper. In these small patches the backbones of P3OT packed parallel to each other and form a lot of hairpin folds. However, these small patches are not stable, desorption, translation and reorientation happen frequently as indicated by the white, red and blue arrows in the images, respectively. On the bare surface of SLG-copper, hexagonal Moiré patterns with period of ~2.5 nm can be clearly revealed, indicating the underneath facet is Cu(111). As time goes on, more and more P3OTs adsorb onto the surface, though desorption of already adsorbed P3OT also happens simultaneously, as indicated by the white arrow in Fig. 1a. Similar as on the HOPG surface, P3OT crystallizes on SLG-copper along three principle orientations, implying that graphene plays a role in determining the orientation of P3OT adlayer. A characteristic of adsorption of P3OT on SLG-copper is that the step edges of underneath copper substrate do not interrupt the growth of P3OT adlayer, P3OT domains can grow across not only monatomic but also multi-atomic step edges with big height difference (Fig. 1c,d). After adsorption of P3OT, most monatomic step edges are hardly distinguishable, in this sense, adsorption of P3OT helps to “fill up” the surface corrugation of SLG-copper.

**Figure 2:**
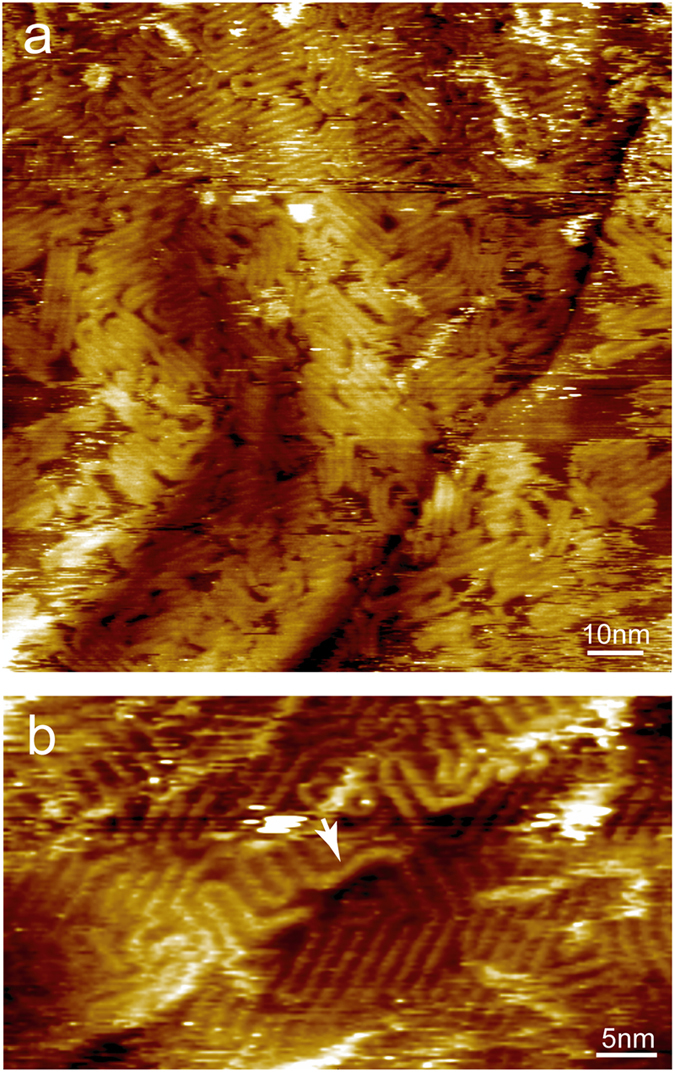
Large scale (a) and high resolution (b) STM images obtained after P3OT covers almost the entire surface, randomly orientated P3OT backbones can be observed on top of the first adlayer. In (**b**), the white arrow points to a P3OT backbone running along a step edge of underneath copper lattice. V_bias_ = 1.2 V, I_set_ = 30 pA.

About one hour after deposition, P3OT can cover almost the entire surface of SLG-copper, even randomly adsorbed second layers can be observed (Fig. 2). In the high resolution image (Fig. 2b), P3OT backbones adsorbed along the underneath step edges can be seen. The orientation of these P3OT backbones does not follow the three main orientations determined by the epitaxial growth. After run along the step edges for a certain distance, these P3OT chains can adsorb continuously across the step edges. Since at the step edges there exist dangling bonds on the copper atoms, the copper-graphene interaction can be stronger at these sites, thus affects the P3OT-graphene interaction, lead to adsorption along the step edges.

**Figure 3:**
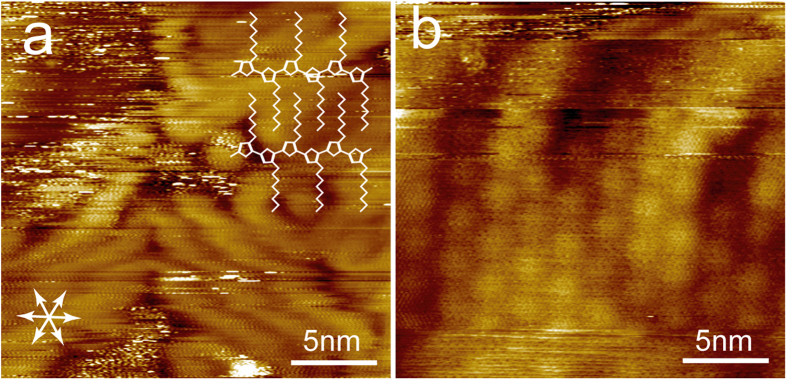
High resolution images of P3OT adsorbed on SLG-copper (a). A schematic model is shown in the upper right corner. The main symmetry axes of graphene are indicated by white arrows. (**b**) An atomic resolution image of the graphene lattice at the same position of (**a**), which reveals clearly the step edges of Cu (111) and a superlattice with a 2.5 nm period. V_bias_ = 1.2 V, I_set_ = 30 pA for (a), V_bias_ = 20 mV, I_set_ = 500 pA for (**b**).

According to the literature, after annealing of the polycrystalline copper foil, low index facets, Cu (111) and Cu (100) are dominant facets^12^,^13^,^14^. Different superlattices were revealed on these two facets after growth of monolayer graphene^12^,^13^. However, our STM observation confirms that the orientation of P3OT domain is determined by the epitaxial adsorption of P3OT on graphene, while the orientation of underneath copper lattice has neglectable influence. As shown in Figure S1, on SLG-Cu (100), the P3OT domains also exhibit three orientations with an angle of 120° between each other. No domains orientated perpendicular to each other were observed. By comparing the high resolution images of P3OT assembly and graphene lattice, the relative orientation of P3OT backbones with respect to graphene lattice can be determined to be perpendicular with respect to one of the main symmetry axes <1 1 −2 0> of graphene lattice (Fig. 3). This is similar as that on HOPG surface. As mentioned above, the monatomic step edges revealed on the atomically resolved graphene lattice shown in Fig. 3b are hardly visible after adsorption of P3OT (Fig. 3a).

**Figure 4:**
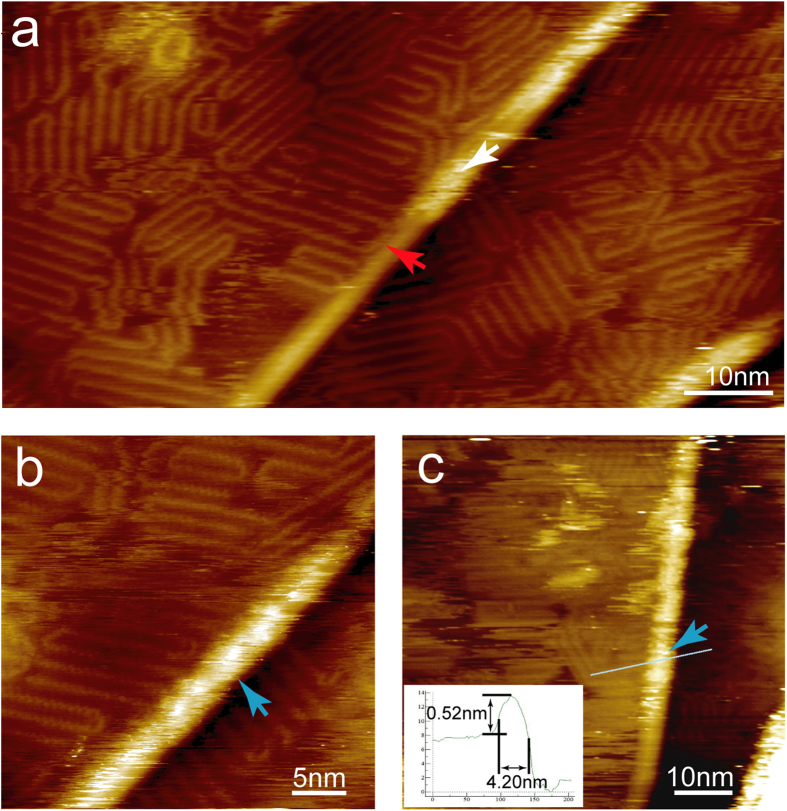
STM images show the adsorption of P3OT on flat terraces and ripples. A ripple with height ~0.5 nm cross the image and shows unstable adsorption of P3OT. The white arrow points to unstably adsorbed P3OT molecules, while the blue arrows indicate P3OT chains with molecular resolution. P3OT chains forming hairpin folds when met the ripple are indicated by the red arrow. V_bias_ = 1.2 V, I_set_ = 30 pA.

During CVD growth of graphene on copper foil, defect-like wrinkles and ripples tend to develop due to strain relief either along the boundaries between crystalline and noncrystalline areas or on step edges of crystalline areas^12^. These structures show significant effect on the adsorption of P3OT, similar as on other small molecule organic semiconductors^8^,^9^,^10^,^11^. As shown in Fig. 4, P3OT can either climb over the ripple or form hairpin folds, as indicated by the white/blue and red arrow, respectively. The P3OT chains can cross over the ripples with different orientations with respect to the ripple axis (Fig. 4b,c). However, the STM characterization indicates that the interaction between P3OT and graphene on such ripples is significantly weaker than on the flat terraces, since the P3OTs adsorbed on the ripples can be easily removed by tip scanning. On the flat terraces the P3OT chains crystallized into close packed domains with the backbone aligned parallel to each other, and the inter-chain distance measures ~1.7 nm. This distance is consistent with that expected for P3OT adsorb with the backbone parallel with the graphene surface and octyl groups orientated perpendicular with respect to the P3OT backbone and fully interdigitate with those from the neighbouring molecules. This is also consistent with above mentioned orientation of P3OT backbone (perpendicular with one of the main symmetry axes of graphene). A schematic model is overlaid on the STM image in Fig. 3a. In the crystalline domains the P3OT backbone adapts all *trans* conformation, while *cis* conformation is a prerequisite for forming folds. When adsorbed on the ripple, the inter-chain distance is significantly larger, (2.63 nm *vs* 1.70 nm), suggesting that the inter-chain interaction is weaker. This is consistent with the observation that P3OT molecules adsorbed on ripples are unstable, and also in accordance with the previous observation of enlarged inter-chain distance on the curvature surface of single wall nanotubes^25^. Similar as on HOPG surface, hairpin and 120° folds are frequently observed in the P3OT adlayer (Fig. 4a), with 60° folds also observable, though very rare^20^,^21^.

In the high resolution images, periodic modulations along the P3OT backbone can be clearly distinguished (Fig. 4b). After careful calibration against the graphene lattices, the periodicity can be determined to be 0.74 ± 0.03 nm, consistent well with that expected for a bi-thiophene repeating unit calculated in gas phase, while larger than that previously observed in the assembly on HOPG surface (0.68 nm)^20^. Mena-Osteritz *et al.* have attributed the observed compression to the epitaxial effect^20^. However, in our work this kind of compression was not observed on SLG-copper surface. Based on the relative orientation of the P3OT backbone and the inter-chain distance, we expect that the octyl chains orientate perpendicular with respect to the thiophene backbone. According to the interdigitated model, the inter-chain distance between octyl groups is 0.37 nm, even smaller than requested for a perpendicularly adsorbed alkane chain^26^. Thus we suppose some of the octyl chains are not adsorbed on the surface.

The experimental results showed that the interaction of P3OT on ripples is weaker than that of P3OT on planar graphene. To verify the intrinsic interfacial interactions between P3OT and graphene or ripples, the adsorption capacity of a model oligomer of poly P3OT on a single layer graphene and ripples were respectively studied using the first principle calculations. For the calculations, the model molecule, P3MT composed of four 3-methyl thiophenes were used to model the P3OT, a piece of graphene with 370 carbon atoms was adopted to simulate the graphene layer, and single-walled carbon nanotubes (SWCNT(6,6) and SWCNT(8,6)-30) were constructed to simulate the different curvature surface of ripples. All the models are large enough to avoid the edge effect.

**Figure 5:**
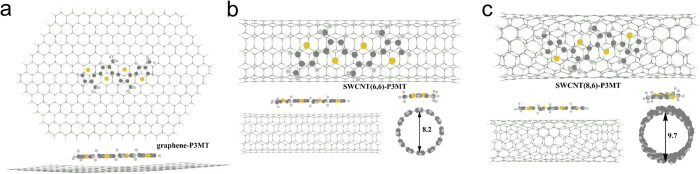
Top and side view of an oligothiophene (P3MT) adsorbed on planar graphene (**a**), SWCNT(6,6) (**b**) and SWCNT(8,6) (**c**).

The calculation results showed that all the most stable conformations of the P3MT on graphene are obtained with sulfur atom above the centre of a six-carbon ring of the graphene or SWCNTs (shown in Fig. 5) due to the polarization. The π-π stacking interaction play a decisive role for the P3MT adsorbed on the graphene or ripples. The adsorption energy on planar graphene (2.62 eV) is c.a. 0.5 eV larger than that of P3MT adsorbed on SWCNTs (2.04 eV for SWCNT(6,6)-P3MT and 2.10 eV for SWCNT(8,6)-P3MT), which confirms the effect of surface curvature on the adsorption of polythiophene.

## Conclusion

To summarize, we have investigated the assembling behaviour of P3OT on the SLG-copper surface. At the solid-liquid interface the adsorption of P3OT on SLG-copper appears quite dynamic. The orientation of P3OT domains is governed by the epitaxial adsorption of P3OT on the three-fold symmetric graphene lattices, while the underlying copper lattice has neglectable effect. The P3OT chains can across even the multi-atomic step edges of underlying copper lattices, however, tend to form hairpin folds when met a ripple along the step edge. The adsorption of P3OTs is weaker on the corrugated ripple surface, even the inter-chain distance is larger, which means that the local curvature also affects the molecule-molecule interaction. The epitaxial adsorption and effect of local curvature were both confirmed by our DFT simulations, which indicates the π-π stacking and polarization play decisive roles in the polythiophene-graphene interaction. The information obtained in this work is relevant to the understanding of graphene-semiconductor interactions which is crucial in many technologically important applications such as molecular electronics, organic solar cells and field-effect transistors.

## Methods

The solvent, 1-phenyloctane (≥99%) and P3OT (regio-regular, M_n_ ~34000) was purchased from Aldrich, and used without further treatment. The graphene was prepared with 25 μm thick copper foils as substrate. The single layer graphene on copper foil (SLG-copper) used here was synthesized by ambient pressure chemical vapor deposition reported elsewhere^27^. Typically, the copper strips (Alfa Aesar 99.95% purity) were soaked in 1 M acetic acid at 42 ^o^C for 10 mins to remove the native oxide and rinsed with deionized water. Then, loaded into fused silica tube and heated to 1035 ^o^C under the H_2_:Ar = 50:450 sccm (standard-state cubic centimetre per minute), followed by annealing for 20 min. Subsequently, 0.1 sccm methane was introduced into the system for 20 min. At last, the furnace was cooled to room temperature under the same gas flow. The SLG-copper was used as prepared for STM substrate.

Samples for STM investigation were prepared by depositing a droplet (~5μL) of the solution of P3OT in phenyloctane (0.1 mg/g) on SLG-copper. STM measurements were performed under room temperature at the liquid-solid interface with a constant current mode (Agilent 5100, USA). Detailed imaging conditions are given in the figure captions. Mechanically cut Pt/Ir (80/20) tips were used. The STM images of the adlayers were corrected for XY drift against SLG-copper lattice.

All geometry optimizations of the P3MT adsorbed on graphene and SWCNTs were respectively carried out using the density functional theory (DFT) method with the SVWN functional^28^,^29^,^30^,^31^. The 3-21G basis set was used for the carbon and hydrogen atoms, the 3-21G* basis set was used for sulfur atom. All calculations were performed using the Gaussian 03 program package^32^. The structures were plotted with Gaussview^33^.

## Additional Information

**How to cite this article**: Jiang, Y. *et al.* The Assembling of Poly (3-Octyl-Thiophene) on CVD Grown Single Layer Graphene. *Sci. Rep.* **5**, 17720; doi: 10.1038/srep17720 (2015).

## Supplementary Material

Supplementary Information
