# Supplementary material for: The Assembling of Poly (3-Octyl-Thiophene) on CVD Grown Single Layer Graphene
Source: Sci Rep. 2015 Dec 4;5:17720. doi: 10.1038/srep17720 (PMC4669485; doi:10.1038/srep17720)
Supplement: Supplementary Information [file srep17720-s1.pdf]

Supporting Information

The Assembling of Poly (3-Octyl-Thiophene) on CVD Grown Single Layer Graphene

Yanqiu Jiang[a], Ling Yang[a], Zongxia Guo[b], Shengbin Lei[a]*

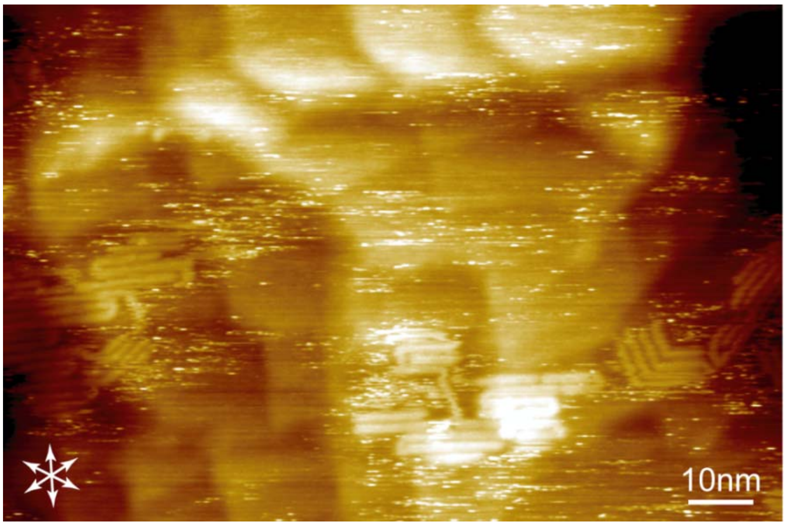

Figure S1. P3OT patches adsorbed on SLG-copper. From the orientation of the step edges, the underneath copper lattice can be determined to be Cu(100). The arrow heads in the lower left corner indicate the main symmetry axes of graphene lattices. The P3OT backbones in the patches are orientated perpendicular with respect to one of the three main symmetry axes. V_{bias} = 1.2 V, I_{set} = 30 pA.

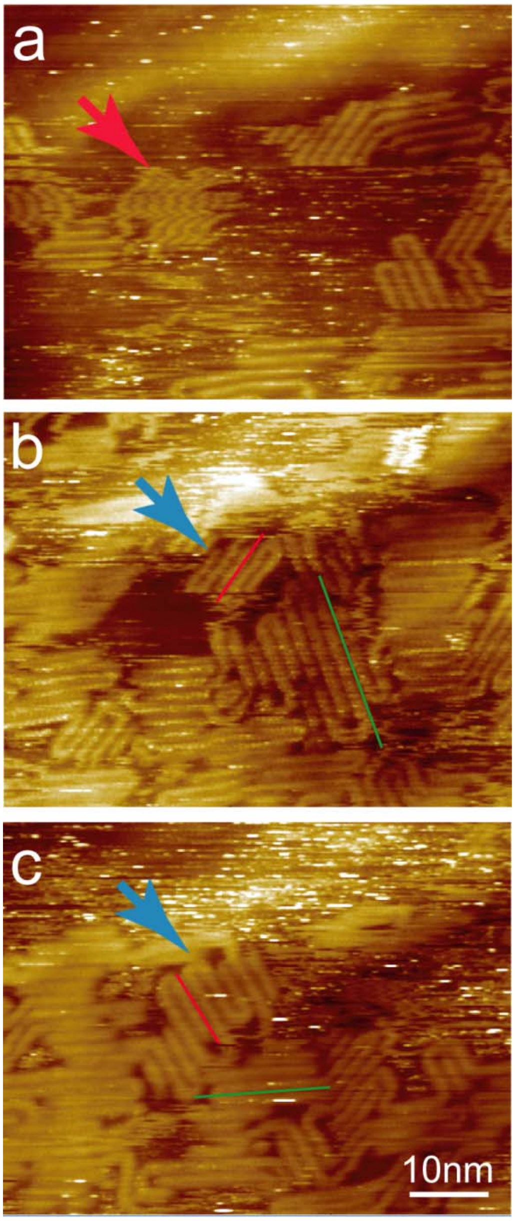

Figure S2. Partial enlargement of Figure 1. The orientations of the P3OT domains are highlighted with the red and green lines.

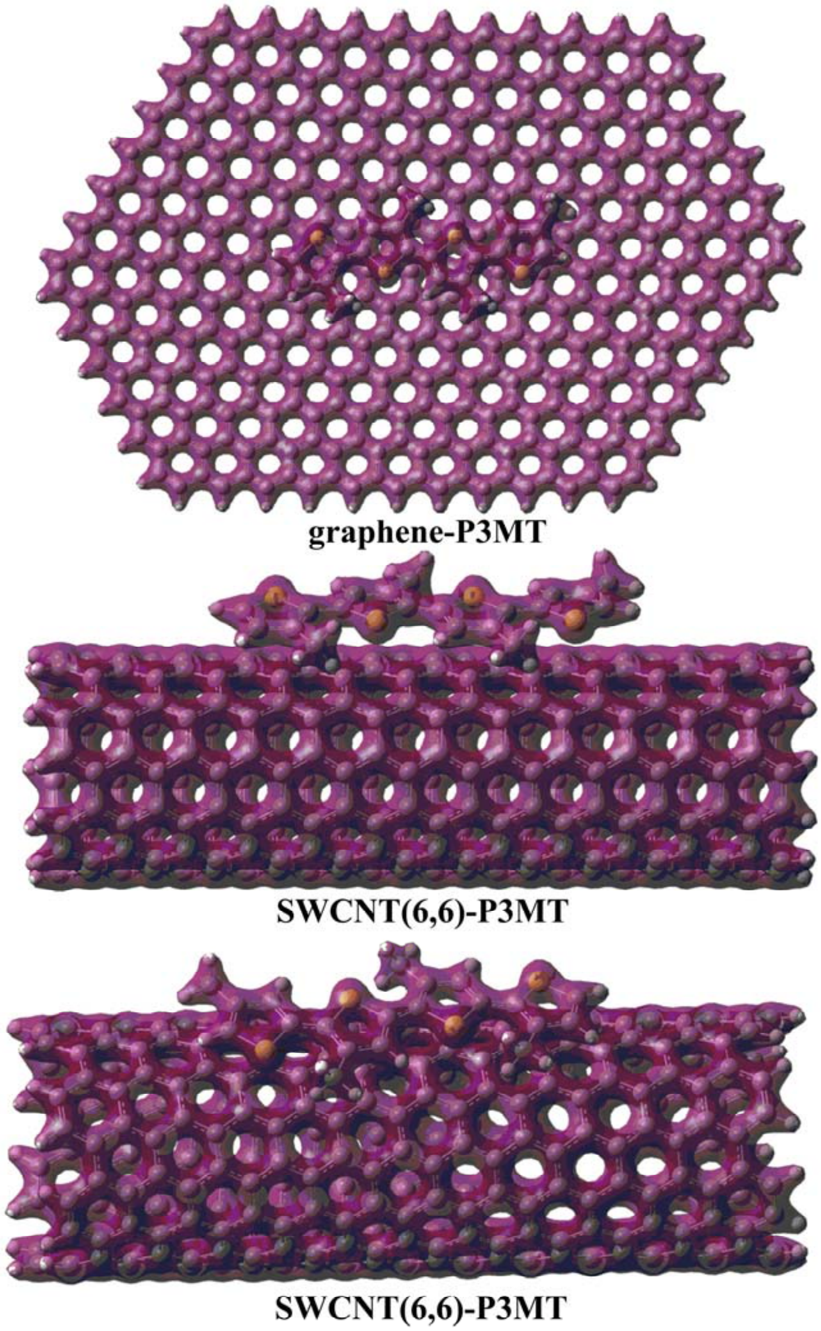

Figure S3. Total electron density calculated and plotted using Gaussview with isovalue equal 0.1. In all the three cases, the electron rich sulfur atoms in the P3MT located on top of the centre of six-member ring of graphene and SWCNTs, highlighting the role of polarization in the interaction between polythiophene and graphene.
